# Journal club in an ICU: rate and factors associated with practice-changing articles. Analysis of 1712 articles read over a 13-year period (2007–2019)

**DOI:** 10.1186/s13613-020-00688-2

**Published:** 2020-06-01

**Authors:** Damien Contou, Marina Thirion, Olivier Pajot, Gaëtan Plantefève, Hervé Mentec

**Affiliations:** Service de Réanimation Polyvalente et Unité de Surveillance Continue, Centre Hospitalier Victor Dupouy, 69, rue du Lieutenant-Colonel Prud’hon, 95100 Argenteuil, France

**Keywords:** Journal Club, Medical Journal, Continuing medical education, Intensive Care, Practice-changing article

Every day more than 2500 original articles are newly indexed in PubMed. Coping with this abyssal amount of medical information is challenging for physicians [1]. Clinicians must read the journals publishing studies having an impact on their daily clinical practices. Our Intensive Care Unit (ICU) has been running a Journal Club (JC) for many years. We aimed at assessing the proportion of practice-changing articles being analyzed during our JC meetings and at identifying factors associated with practice-changing articles.

From August 2007 to August 2019, we prospectively collected the references of articles presented at each JC meeting. Our medical–surgical ICU has 18 beds (12 intensive and 6 intermediate care beds) and our 760-bed hospital is university-affiliated. Our medical team comprises 4 attending intensivists, 3 fellows in intensive care and 7 residents. JC sessions are scheduled weekly and last from 1.5 to 2.5 h depending on the number of participants exposing an article (one article per participant). Participants are free to choose and expose orally an article recently published in any medical journal (general, ICU or non-ICU specialized). Presentation of an article lasts up to 10 min and is followed by a 5-min discussion. Most often, only the participant who reports an article had read the article before the JC meeting. The number of articles published during the study period was determined with PubMed. All the exposed articles were independently reviewed by two of us (DC and HM) and were considered to change practices when at least one considered they did (did this article change my daily clinical practice?)

During the study period, 313 JC meetings were held and 1712 articles from 97 journals were exposed. Median number of articles discussed per meeting was 6 [4–7]. Median number of physicians attending each JC meeting was 7 [6–8] (attendings: 2 [2–3], fellows: 1 [1–2], residents: 3 [2–3]). After removing duplicates and non-original articles, 1568 unique original articles were exposed, accounting for 0.01% (95% CI [0.01–0.02%]) of the 10.982.188 original articles referenced in PubMed during the same period. General, ICU and non-ICU specialized journals accounted for 32%, 47% and 21% of the exposed articles, respectively. Compared to general and non-ICU specialized journals, the proportion of read-over-published articles was higher for ICU specialized journals (0.18% vs. 0.13% vs. 2.61%, respectively; *p* < 0.0001).

Only 93/1568 (5.9%) articles were considered as practice-changing. The two reviewers agreed on 95% of the evaluations. Factors associated with practice-changing articles were identified by univariable analysis and were as follows: age of the physician reporting the article (attending 7% vs. fellow 7% vs. resident 4%; p = 0.03) and type of medical journal in which the article was published (general 9% vs. ICU specialized 4% vs. non-ICU specialized 5%; p = 0.001). The year of publication of the article and the specialty of the physician reporting the article were not associated with practice-changing articles. Data regarding the 5 most read general, ICU and non-ICU specialized journals, accounting for 87% of the unique original articles exposed during our JC meetings, are detailed in Table 1.

**Table 1:** Number of original articles published from August 2007 to August 2019 in the 5 most read journals during our journal club meetings in the 3 journal categories, percentages of articles exposed during our journal club meetings and percentages of practice-changing articles

Journals (*n* = 97)	Nb original articles published	Nb original articles read	% [95% CI] read/published articles	Nb of original practice-changing articles	% [95% CI] of original practice-changing read articles
***General journals (n = 15)***	***294,167***	***495***	***0.17 [0.15–0.18]***	***45***	***9.09 [6.86–11.95]***
*New England Journal of Medicine*	8534	215	2.52 [2.21–2.87]	25	11.63 [8.00–16.60]
*Journal of the American Medical Association*	8678	135	1.56 [1.32–1.84]	9	6.67 [3.55–12.18]
* The Lancet*	10,426	71	0.68 [0.54–0.86]	9	12.68 [6.81–22.37]
* British Medical Journal*	12,488	21	0.17 [0.11–0.26]	0	0.00 [0.00–15.46]
* Archive of Internal Medicine*	1653	19	1.15 [0.74–1.79]	0	0.00 [0.00–16.82]
Others (*n* = 10)	252,388	34	0.01 [0.01–0.02]	2	5.88 [1.63–19.09]
***ICU specialized journals (n = 12)***	***29,553***	***728***	***2.46 [2.29–2.65]***	***31***	***4.26 [3.02–5.98]***
* Critical Care Medicine*	4927	361	7.33 [6.63–8.09)	15	4.16 [2.53–6.74]
* Intensive Care Medicine*	3014	200	6.64 [5.80–7.58]	7	3.50 [1.71–7.05]
* American Journal of Respiratory and Critical Care Medicine*	4926	77	1.56 [1.25–1.95]	3	3.90 [1.33–10.84]
* Critical Care*	4268	57	1.34 [1.03–1.73]	4	7.02 [2.76–16.70]
* Annals of Intensive Care*	692	16	2.31 [1.43–3.72]	1	6.25 [1.11–28.33]
Others (*n* = 7)	11,726	17	0.14 [0.09–0.23]	1	5.88 [1.05–26.98]
***Non-ICU-specialized journals (n = 70)***	***264,429***	***345***	***0.13 [0.12–0.14]***	***17***	***4.93 [3.10–7.75]***
* Clinical Infectious Diseases*	7661	76	0.99 [0.79–1.24]	7	9.21 [4.53–17.81]
* Chest*	5102	65	1.27 [1.00–1.62]	2	3.08 [0.85–10.54]
* Anesthesiology*	3613	23	0.64 [0.42–0.95]	1	4.35 [0.77–20.99]
* The Lancet Infectious Diseases*	2369	18	0.76 [0.48–1.20]	1	5.56 [0.99–25.76]
* Annals of Surgery*	4259	14	0.33 [0.20–0.55]	1	7.14 [1.27–31.47]
Others (*n* = 65)	241,425	149	0.06 [0.05–0.07]	5	3.36 [1.44–7.61]
***Original articles published in journals read at least once during our journal club meetings***	***588,149***	***1568***	***0.27 [0.25–0.28]***	***93***	***5.93 [4.87–7.21]***
***All original articles referenced on PubMed***	***10,982,188***	***1568***	***0.01 [0.01–0.02]***	***93***	***5.93 [4.87–7.21]***

*Nb* number, *CI* confidence interval

The proportion of articles exposed during our JC meetings appears paltry (0.01%) compared to the huge amount of literature published every year. Only 5.9% of the exposed articles were considered as practice-changing. Similarly, it was recently reported that among 1240 articles assessing the impact of any intervention on mortality of ICU patients, only 27 showed a reduction, i.e., 2.2% [2]. General journals appear to publish a higher proportion of practice-changing articles compared to ICU or non-ICU-specialized journals. Indeed, the 27 articles reported as decreasing mortality of ICU patients were more often published in general journals (55%) than in ICU-specialized (30%) or non-ICU-specialized (15%) journals [2].

Limitations of our study include that physicians did not read articles only for JC and may read more than one article to prepare JC. Therefore, our data underestimate the real figure. Moreover, all the articles exposed during our JC meetings did not focus only on the ICU practices, potentially responsible for an underestimation of the rate of practice-changing articles. It is also likely that JC participants were more prone to choose articles considered as practice-changing, and this selection bias overestimates the clinical impact of medical journals. Last, practice-changing was subjectively assessed by only two of us limiting generalizability.

In a French university-affiliated ICU with regular JC meetings, exposed articles were a drop in the ocean of medical literature and the proportion of practice-changing articles appeared minor. Of course, other sources of continuing medical education can be used [3], but it is also possible to increase the profitability of JC by following published recommendations [4].

## Data Availability

The dataset used and analyzed for the current study is available from the corresponding author on reasonable request.

## Abbreviations

ICU: Intensive Care Unit
JC: Journal Club
